# Full-Genome Sequence of a Reassortant H1N1 Swine Influenza Virus Isolated from Pigs in Italy

**DOI:** 10.1128/genomeA.00778-13

**Published:** 2013-10-03

**Authors:** Chiara Chiapponi, Laura Baioni, Andrea Luppi, Ana Moreno, Alberto Castellan, Emanuela Foni

**Affiliations:** OIE Reference Laboratory for Swine Influenza, Istituto Zooprofilattico Sperimentale della Lombardia ed Emilia Romagna, Parma, Italya; Istituto Zooprofilattico Sperimentale della Lombardia ed Emilia Romagna, Reggio Emilia, Italyb; Istituto Zooprofilattico Sperimentale della Lombardia ed Emilia Romagna, Virologia Brescia, Italyc; DVM, Vicenza, Italyd

## Abstract

In this study, the full-genome sequence of a novel reassortant H1N1 swine influenza virus (SIV) is reported. The isolate has a hemagglutinin (HA) gene of the pandemic H1N1 influenza virus, but it carries the seven genome segments of the avian-origin H1N1 SIV currently circulating in European pig farms.

## GENOME ANNOUNCEMENT

Influenza A viruses (IAVs) are members of family *Orthomyxoviridae*, genus *Influenzavirus A*, which are classified into 17 hemagglutinin (HA) and 10 neuraminidase (NA) based on the antigenicity of these surface glycoproteins (1, 2). Nowadays, there are three principal subtypes of IAVs circulating in the Italian pig population: the avian-like H1N1 and the human-derived H1N2 and H3N2 lineages (3); recently, H1N1pdm2009 has also been described (4). In addition, some reassortants between these lineages have sporadically been detected (5, 6), but only the H1N2 human-derived lineage became established in the Italian pig population (7). Reassortant viruses carrying genome segments from the pandemic H1N1 are reported in many European countries, including Germany (8, 9), United Kingdom, and Hungary (10, 11). In 2010, a reassortant between H1N1pdm and the H1N2 human-derived lineage was isolated from pigs in Italy (7). We report the complete genome sequence of a swine influenza virus (SIV) isolated from nasal swabs collected during an outbreak of respiratory disease in 60-day-old pigs in a farm, in Treviso province, where the cocirculation of avian-like H1N1 and H1N1pdm swine influenza viruses had been detected during the previous 6 months. Viral RNA was extracted from cell-cultured virus, and the sequences of full-genome segments were obtained on a MiSeq platform (Illumina) as described previously (12). The data were *de novo* assembled on BaseSpace Cloud (Illumina) by the DNAStar application and were analyzed by the Lasergene package software (version 10.1.2). The eight genome segments encode 12 proteins: polymerase basic 2 (PB2) (759 amino acids [aa]), PB1-F1 (757 aa), PB1-F2 (76 aa), polymerase acidic (PA) (716 aa) and PA-X (252 aa), HA (566 aa), nucleoprotein (NP) (498 aa), NA (469 aa), M1 (252 aa) and M2 (97 AA), and nonstructural 1 (NS1) (217 aa) and NS2 (61 aa). The predicted HA protein showed K at residue 147 (H1 numbering), which is associated with the stabilization of the interaction between HA and sialic acid (13). No mutation in D222 (14) (H3 numbering) was observed. No resistance markers for oseltamivir (H275Y, N295S) or zanamivir (Q136K, K150T, I223R) were observed (15, 16) in the NA protein. NS1 is a truncated protein of 217 aa similar to that in recent Italian isolates (7). The amantadine resistance markers S31N, R77Q, and V27A (17) were observed in the M2 protein. Interestingly, the PB1 gene encodes an infrequently occurring truncated protein PB1-F2 of 76 aa. Phylogenetic analyses showed the relatedness of the HA gene with those of the 2009 pandemic strains (up to 98% nucleotide identity with the reference strains), including the pandemic Italian porcine (4) and human isolates, whereas the NA and the internal genes were found to be more similar to Italian and some other Eurasian avian-derived H1N1 SIV strains (97 to 98% nucleotide identity). These findings suggest that strain H1N1 evolved from the pandemic H1N1 strain and an avian-like H1N1 pig strain through natural reassortment.

### Nucleotide sequence accession numbers.

The genome sequence of A/Swine/Italy/73449/2013/H1N1 has been deposited in GenBank under accession no. KF500932 to KF500939.
